# The localization of molecularly distinct microglia populations to Alzheimer's disease pathologies using QUIVER

**DOI:** 10.1186/s40478-023-01541-w

**Published:** 2023-03-18

**Authors:** Ryan K. Shahidehpour, Abraham S. Nelson, Lydia G. Sanders, Chloe R. Embry, Peter T. Nelson, Adam D. Bachstetter

**Affiliations:** 1 Spinal Cord and Brain Injury Research Center, University of Kentucky, 741 S. Limestone St., Lexington, KY 40536 USA; 2 Department of Neuroscience, University of Kentucky, Lexington, KY 40536 USA; 3 Sanders-Brown Center On Aging, University of Kentucky, Lexington, KY 40536 USA; 4 Division of Neuropathology, Department of Pathology and Laboratory Medicine, University of Kentucky, Lexington, KY 40536 USA

**Keywords:** Multiplexed tissue imaging, Single-cell analysis, Digital pathology, Histology, Neurodegenerative disease, Glia, Neuropathology

## Abstract

New histological techniques are needed to examine protein distribution in human tissues, which can reveal cell shape and disease pathology connections. Spatial proteomics has changed the study of tumor microenvironments by identifying spatial relationships of immunomodulatory cells and proteins and contributing to the discovery of new cancer immunotherapy biomarkers. However, the fast-expanding toolkit of spatial proteomic approaches has yet to be systematically applied to investigate pathological alterations in the aging human brain in health and disease states. Moreover, post-mortem human brain tissue presents distinct technical problems due to fixation procedures and autofluorescence, which limit fluorescence methodologies. This study sought to develop a multiplex immunohistochemistry approach (visualizing the immunostain with brightfield microscopy). Quantitative multiplex Immunohistochemistry with Visual colorimetric staining to Enhance Regional protein localization (QUIVER) was developed to address these technical challenges. Using QUIVER, a ten-channel pseudo-fluorescent image was generated using chromogen removal and digital microscopy to identify unique molecular microglia phenotypes. Next, the study asked if the tissue environment, specifically the amyloid plaques and neurofibrillary tangles characteristic of Alzheimer's disease, has any bearing on microglia's cellular and molecular phenotypes. QUIVER allowed the visualization of five molecular microglia/macrophage phenotypes using digital pathology tools. The recognizable reactive and homeostatic microglia/macrophage phenotypes demonstrated spatial polarization towards and away from amyloid plaques, respectively. Yet, microglia morphology appearance did not always correspond to molecular phenotype. This research not only sheds light on the biology of microglia but also offers QUIVER, a new tool for examining pathological alterations in the brains of the elderly.

## Introduction

Histology on preserved human brain specimens is a robust method to visualize pathology for clinical diagnosis and experimental investigations of neuropathological changes in Alzheimer’s disease (AD). A rapidly expanding body of evidence demonstrates the central role of microglia in neurodegenerative diseases [1–4]. A consensus statement also highlighted the need to consider the importance of the brain environment when describing microglia phenotypes and not an over-simplified assessment of microglia (i.e., M1 vs. M2 or resting vs. activated) [3]. Therefore, integrating microglial cells’ morphological, molecular, and spatial phenotypes is needed to advance the field.

The recent development of spatial proteomics has revolutionized the study of tumor microenvironments in oncology by providing multidimensional single-cell (and subcellular structural) level analyses of protein expression while maintaining the spatial context of the microenvironment [5, 6]. Defining immune cell spatial interactions in the tumor microenvironment provides emerging prognostic and predictive biomarkers for cancer immunotherapy [7–10]. As the technique matures, it is predicted to be a clinically important tool for cancer and will be essential for understanding pathology in neurodegenerative diseases [5, 6].

Techniques such as flow cytometry, single-cell mass cytometry (cytof), and single-cell RNA sequencing (scRNA-seq) provide remarkable depth in characterizing cellular status for microglia and other cell types [11, 12]. However, these tissue-level techniques lose much of the single-cell level information of microglia’s interactions within the brain microenvironment and the morphological appearance of the cell. A conventional microscopy method, on the other hand, provides spatial information but does not allow visualization and quantification of cells classified according to complex phenotypic marker combinations. To advance the understanding of microglial function in the aged human brain, approaches such as the spatial proteomics used to define immune profiles in the tumor microenvironment are needed to characterize cellular inter-relationships in brains and positional proximities with neuropathological changes.

Commercial methods of spatial proteomics have been recently developed, such as Lunaphore, Akoya biosciences Phenocycler (CODEX), or NanoString’s GeoMx, which provides high-plex spatial imaging of 100+ RNA or protein markers [6, 13]. However, these methods may require expensive specialty antibodies (rare mental conjugated antibodies), specialty equipment or microscopes, and novel methodologies requiring specialized training, and limited compatibility with formalin-fixed and paraffin-embedded (FFPE) tissue. In addition, subcellular spatial proteomic methods have been described, which build on the well-established immunostaining technique [5, 6, 14]. In these techniques, an iterative approach is used if many proteins are to be visualized where the same tissue is stained, imaged, and then re-stained with these techniques using successive antibody or label detection rounds. Then computational methods are applied for image co-registration and visualization of the staining using colorimetric and fluorescence visualization methods [5, 6]. Colorimetric visualization methods are useful in postmortem human brain tissue, because of the high levels of autofluorescence in the tissue.

This project’s goal was to develop a multiplex immunostaining method that could spatially profile proteins, specifically in FFPE human brain tissue. Our focus was on the microglial cells’ molecular signature in relation to neurodegenerative disease pathology. We generated the Quantitative multiplex Immunohistochemistry with Visual colorimetric staining to Enhance Regional protein localization (QUIVER) method to overcome challenges of immunostaining in human FFPE tissue, including autofluorescence and limitation of the same primary antibody host species. With QUIVER, we sought to use conventional immunohistochemistry (IHC) techniques and equipment to expand the reach of spatial proteomics to more researchers. Within, the results of QUIVER using a nine-antibody panel provide a spatial image analysis of microglia subpopulations in the presence of amyloid and tau pathology.

## Methods

### Human subjects

**Fig. 1:**
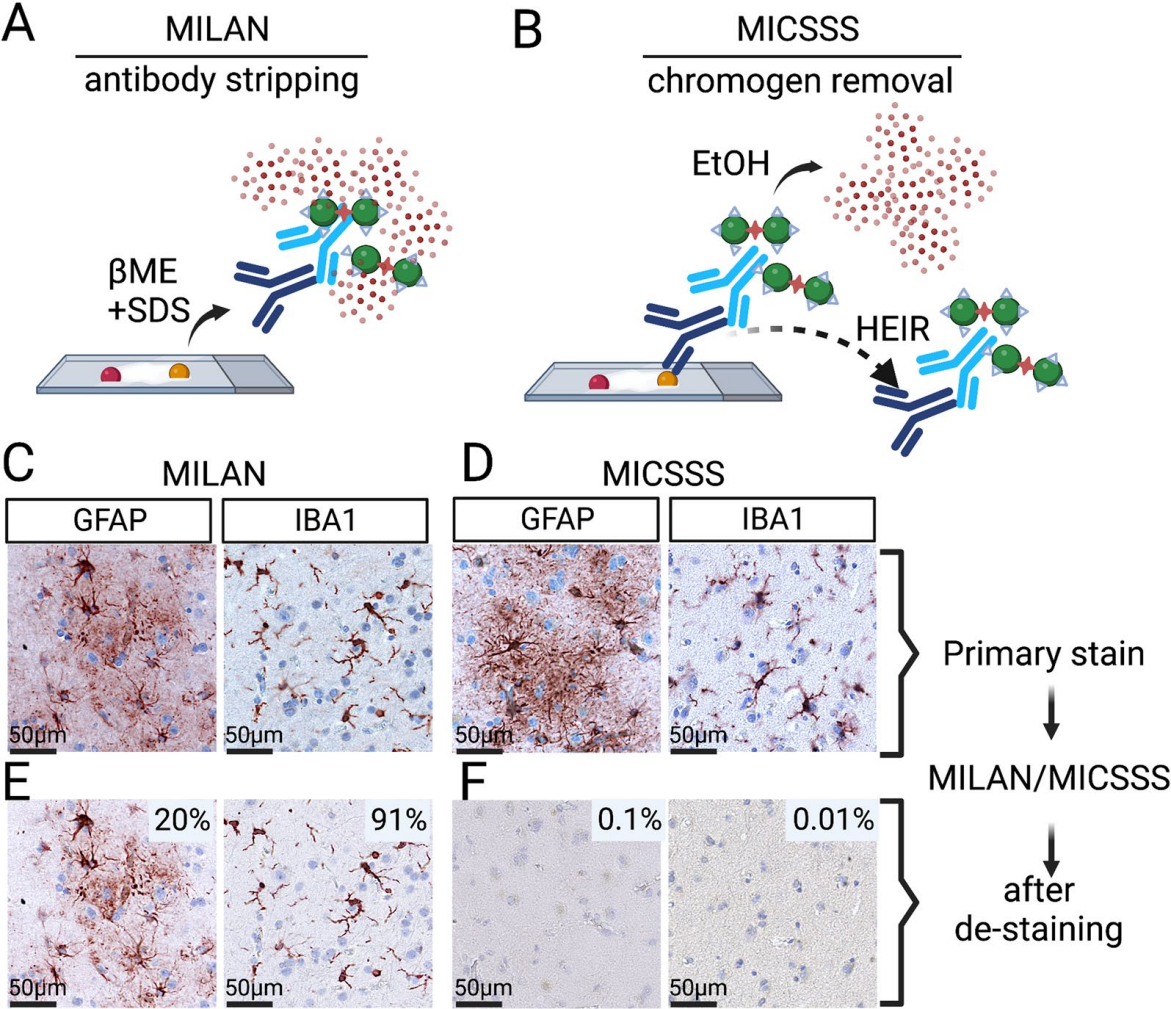
Comparison of two mIHC protocols for use in human FFPE tissue. (**A**) The multiple interactive labeling by antibody neodeposition (MILAN) method uses β-mercaptoethanol and sodium dodecyl sulfate (βME + SDS) to strip the antibody complex. (**B**) The multiplexed immunohistochemical consecutive staining on a single slide (MICSSS) uses ethanol (EtOH) to wash out the chromogen. Heat-induced epitope retrieval (HIER) is predicted to elute the antibody partially. (**C**, **D**) GFAP and IBA1 were stained on serial sections of human FFPE brain tissue. Following the MILAN or MICSSS procedure the slide were re-imaged. (**E**) 20% of GFAP staining and 90% of IBA1 staining were found across the entire tissue section following the MILAN method. (**F**) The MICSSS method effectively reduced the re-development of GFAP and IBA1 to less than 0.1%

**Fig. 2:**
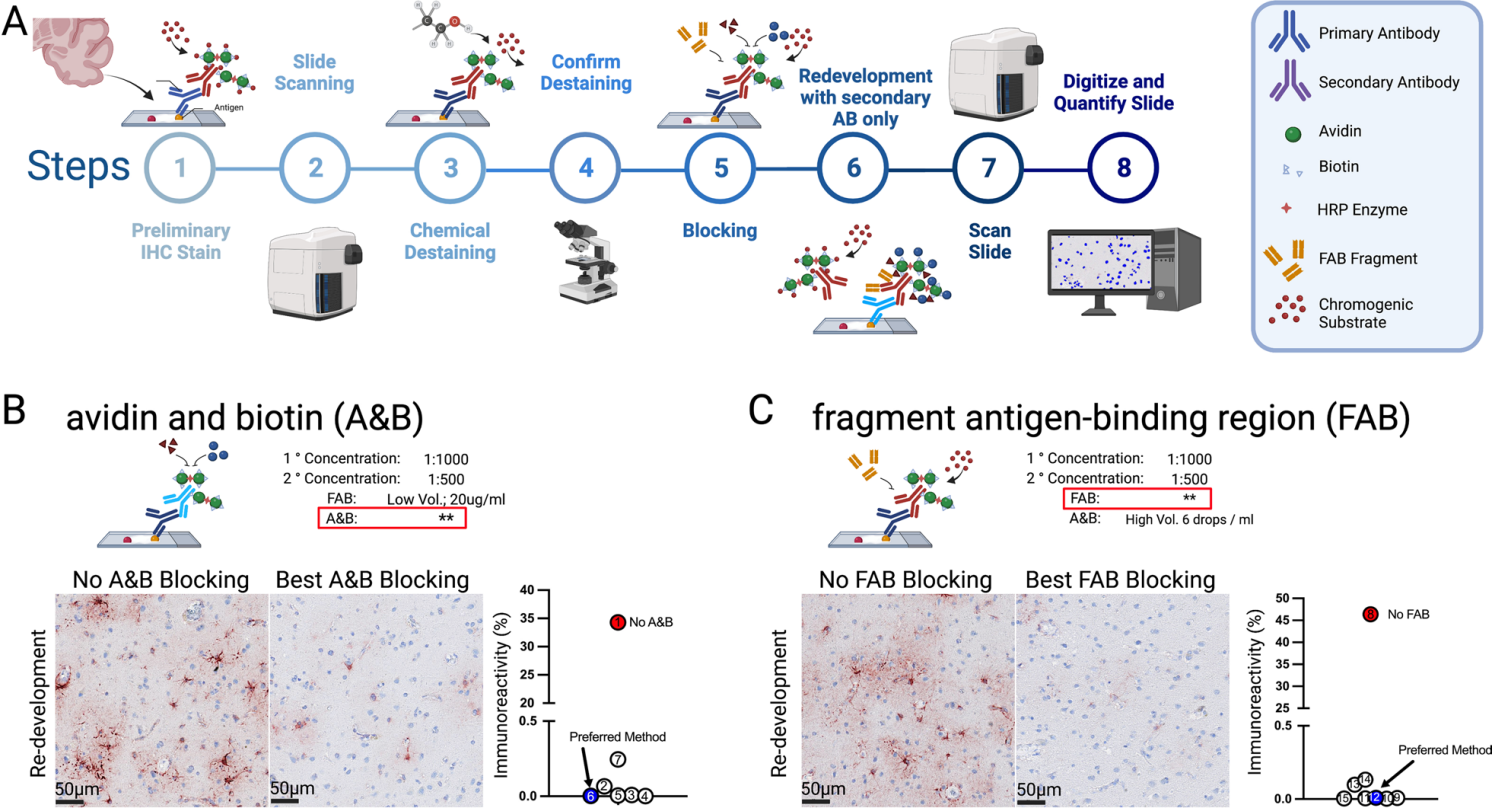
Blocking step optimization to limit antibody cross-reactivity. (**A**) Experimental workflow for antibody cross-reactivity test: (1) Indirect IHC is completed using an ethanol-soluble chromogen. (2) The digital slide is generated using a slide-scanning microscope. (3) The chromogen is removed using the chemical de-staining method. (4) The slide is then visually inspected to confirm the de-staining was ~ 100% efficient. (5) Additional blocking steps are added to limit cross-reactivity. (6) The tissue is stained following step 1, omitting the primary antibody. (7) A digital slide is created, and (8) digital pathological tools are used to quantify the percentage of cross-reactivity by co-registration and direct comparison of the image from step 2 to the image in step 8. (**B**) Avidin and biotin (A&B) blocking conditions were tested (Table 2). The photomicrographs show a comparison of the GFAP staining between the best A&B block condition versus the omission of the A&B blocking step. Digital neuropathological quantification of the area fraction of GFAP^+^ staining show that approximately 35% of the staining remains if the A&B blocking step is omitted. (**C**) The effect of varying the FAB blocking conditions (Table 2) were tested while holding the best A&B blocking condition from B constant. A high degree (46%) of GFAP^+^ staining was seen in samples lacking the FAB blocking step following re-staining whit the secondary antibody alone. By area fraction digital quantification, the degree of re-development of the GFAP staining was further blocked beyond the optimized A&B conditions using a higher concentration of FAB a incubating the samples in a greater volume of the blocking solution

Human brain tissue samples comprising the superior mid-temporal gyrus (Brodmann areas 21/22) were acquired from the University of Kentucky Alzheimer’s Disease Research Center (UK-ADRC) biobank [12]. Samples were formalin-fixed and paraffin embedded. All patient identifiers were removed, and investigators were blind to case information. Sex of subjects was unknown. Sections in FFPE blocks were cut on a microtome at a thickness of 6 μm and mounted on Superfrost Plus microscope slides (Fisher, 22-037-246) and incubated in a 37° C oven overnight to dry. For digital quantification and analysis, the tissue was compared between entire adjacent sections of sMTG (Figs. 1, 2, 4, and 5). To compare changes in antigenicity, eight cortical punches measuring 1 mm in diameter were taken from various cortical regions in serially sectioned TMA slides. Finally, for quantification and characterization of microglial phenotypes, four sub-regions from throughout the human sMTG were compared (Figs. 7, 8, 9).

### Immunohistochemistry

Sections were deparaffinized and rehydrated in xylene followed by decreasing concentrations of alcohol. Heat-induced antigen retrieval (HEIR) was completed on tissue using pre-warmed citrate buffer (Diva Decloaker (10×), BIOCARE Medical) in a microwave (6 min (power setting 3, 500 watts) followed by cooling for 15 min. Once sections were cooled, they were rinsed in running water. Following antigen retrieval, tissue was permeabilized using 0.4% triton- × 100 for 30 min. Endogenous peroxidases were then quenched in 0.3% H_2_O_2_ in methanol for 30 min. Tissue sections were then incubated for 60 min in blocking buffer (10% normal goat serum, with 0.2% Triton-X 100, in tris buffer saline) at room temperature prior to antibody addition. Sections were incubated in primary antibody overnight in 4 °C. Antibody list may be found in Table 1. A biotinylated secondary antibody specific to each host species was amplified using an avidin–biotin substrate (ABC solution, Vector Laboratories, catalog no. PK-6100), followed by color development in ImmPACT® AMEC Red Substrate (Vector Laboratories, SK-4285). Stained sections were counterstained in hematoxylin and covered using aqueous mounting media (VectaMount® AQ Aqueous Mounting Medium, H-5501-60, (Vector Laboratories). Throughout the staining process, tissue was stained using the Sequenza staining rack (Thermo Fisher #73310017)—a device that uses a capillary action to irrigate mounted sections. For each wash and incubation step, 1 ml of reagent was added to the slide. During the blocking steps, in addition to the Sequenza, slides were also fully submerged in a lockmailer jar that contained 12 ml of reagent and put on a shaker during incubation steps.

**Table 1:** Antibodies used during multiplex staining

Antibody	RRID	Manufacturer	Cat. number	Host species	Concentration
P2Y12	AB_2669027	Atlas	HPA014518	Rabbit	1/500
TMEM119	AB_2681645	Atlas	HPA051870	Rabbit	1/500
Ferritin	AB_259622	Thermo fisher	F5012-2ML	Rabbit	1/1,500
CD45	AB_2750582	Thermo fisher	M070101-2	Mouse	1/200
CD68	AB_2661840	Agilent	GA609	Mouse	1/100
Iba1	AB_2493179	Synaptic systems	234–004	Guinea Pig	1/1,000
GFAP	AB_10013382	Dako	Z0334	Rabbit	1/5,000
PHF-1	–	Gift from Dr. Peter Davies	Mouse	1/500	
Aβ	AB_2533317	Thermo fisher	37–4200	Mouse	1/10,000

### Antibody stripping

To also test the ability to remove primary antibodies from tissue, antigen removal using β-mercaptoethanol/sodium dodecyl sulfate (βME + SDS) was completed as published [15, 16]. Briefly, βME + SDS removal was performed by mixing 20 ml 10% SDS with 12.5 ml 0.5 M Tris–HCl, pH 6.8, and 67.5 ml ultra-pure water. 0.8 ml of βME was added to solution and sections incubated in pre-warmed βME + SDS on shaker for 30 min. Sections were then washed in diH2O followed by TBS-T washes and standard blocking steps before immunostaining.

### Chromogen removal

Tissue was stained using the ABC indirect method and developed using the ImmPACT AMEC Red Substrate kit (Vector Laboratories). Following staining and slide scanning at 20× magnification, the coverslips were removed in water and residual mounting medium was removed by washing sections in 1× PBS for 3× 10 min. To wash out the ImmPACT AMEC Red chromogen (Vector Laboratories), sections were incubated in 50% ethanol (1×, 2 min), 70% ethanol (with 1% HCL) (1×, 2 min), 100% ethanol (1×, 5 min), 70% ethanol (1×, 3 min), and running water (5 min). Following these steps, a coverslip was placed over the rehydrated section and the chromogen removal was evaluated at 20× magnification using a brightfield microscope to confirm a lack of residual staining. The coverslip was floated off the section in PBS and staining protocol commenced.

### Cyclic multiplex immunohistochemistry

Iterative rounds of immunostaining started with the first round of immunohistochemistry, as described above. After the microscope slide scanning, the coverslip was removed by soaking the slide in water, typically overnight on a shaker, until the coverslip fell off. Next, the chromogen was removed as described above. Heat-induced antigen retrieval was then completed as described previously, followed by a series of blocking steps. First, the slides were incubated for 30 min in 0.4% Triton-X 100. Then endogenous peroxidases were quenched with 0.3% H_2_O_2_ in methanol for 30 min. The samples were then incubated for 60 min in blocking buffer (10% normal goat serum, with 0.2% Triton-X 100, in tris buffer saline) at room temperature. Residual avidin and biotin was blocked by incubating in avidin (Vector Laboratories) diluted in blocking buffer for 1 h, washed three times in blocking buffer, and then incubated in biotin (Vector Laboratories) diluted in blocking buffer for 1 h. If the same species primary antibody was used, the samples were incubated in fragment antigen-binding region (FAB) (Jackson Immuno Research labs) overnight at 4 °C. For the remaining steps, we used the immunohistochemistry protocol described above. For each round of staining a no primary antibody control and positive control samples were included.

### Slide scanning and image registration

A Zeiss Axio Scan Z.1 slide scanner was used to image the slide in its entirety at 20× magnification creating a single high-resolution image. After scanning, chromogenic images were loaded into HALO software (Indica labs, version 3.4), and stains were registered and deconvolved to make a single pseudo-fluorescent image. This was achieved by separating the chromogenic stain from the hematoxylin stain using the HALO deconvolution algorithm, which uses color selection and thresholding to create a single-channel image. Then using the HALO Serial Registration module, the multiple rounds of staining were merged into a pseudo-fluorescent image.

### Digital pathological investigations

The object colocalization algorithm in HALO version 3.4 was used to quantify colocalization and the number of cells in stained tissue. By thresholding, the pseudo-fluorescent image for each channel, the Object Colocalization module calculated the number of cells. The computer-generated markup image was used to confirm the specificity of the algorithm. The HALO proximity analysis was used to determine the distance of microglia from Aβ plaques and PHF1^+^ cell bodies. A size exclusion of 100 μm was used when defining the Aβ plaques and PHF1^+^ cell bodies to avoid detecting small diffuse Aβ or PHF1^+^ neurites.

## Results

Two promising methods of iterative colorimetric multiplex IHC (mIHC) were previously described [9, 15]. The multiple iterative labeling by antibody neodeposition (MILAN) method used a striping technique to elute the primary antibody before re-staining using β-mercaptoethanol and sodium dodecyl sulfate (βME + SDS) (Fig. 1A). Removal of the staining antibody complex is an advantage of the MILAN technique, as this would allow for successive rounds of staining with an antibody raised in the same species and eliminates concerns regarding steric hindrance [15]. However, the βME + SDS used to strip the antibody may damage antigenicity for future rounds of staining. In contrast, the multiplexed immunohistochemical consecutive staining on a single slide (MICSSS) uses a method that involves washing out the chromogen using ethanol (EtOH) without antibody elution (Fig. 1B) [9]. Between each round of MICSSS protocol, heat-induced antigen (epitope) retrieval (HIER) is completed, which may partially elute the antibody. Yet, it is known to be ineffective at fully stripping the antibody complex [9]. Thus, the MICSSS approach has a potential limitation of cross-reactivity when the antibody is raised in the same species used in consecutive rounds of staining. While steric hindrance remains a concern with MICSSS, prior evidence suggest that it is uncommon, and may provide useful information regarding neighboring epitopes [9].

Our first approach was to directly compare the MILAN vs. the MICSSS protocols, as neither method has been used in human brain FFPE tissue. We used two antibodies known for robust and reproducible staining in human brain FFPE tissue. The first antibody targets glial fibrillary acidic protein (GFAP), a protein highly expressed by astrocytes [17]. The second antibody targets ionized calcium binding adaptor molecule 1 (IBA1), a pan microglia/macrophage marker [18]. Given the widespread pattern of staining throughout the brain and that staining for both markers is increased with ADNC, we anticipated that these two markers would challenge the two de-staining methods.

To test the MILAN vs. MICSSS protocols serial sections of FFPE human brain tissue were stained with GFAP or IBA1 and imaged using slide scanning microscopy (Fig. 1C, D). The MILAN protocol uses βME + SDS to strip the antibody complex and not soluble chromogen stain. However, we found that after the MILAN protocol ~ 20% of the GFAP staining and ~ 90% of IBA1 staining was still present (Fig. 1E), showing limited efficiency of MILAN protocol for removal of the antigen/antibody complex in human brain sections. In contrast, the MICSSS method which uses an ethanol soluble chromogen was highly effective at with less than 1% of the original stain present after EtOH-mediated chromogen removal (Fig. 1F).

### Refinement of MICSSS blocking steps for use in human brain FFPE tissue

The MICSSS was selected as the framework for our mIHC protocol. An important first step was addressing cross-reactivity issues when the same species-antibodies are used in consecutive rounds of staining. To minimize cross-reactivity in the mIHC protocol, we used an iterative process of staining with rabbit anti-GFAP, de-staining following the MICSSS protocol, and re-staining while omitting the primary antibody and using the same anti-rabbit secondary antibody (Fig. 2). Determining how much staining was present at the end of this procedure would indicate potential cross-reactivity if antibodies raised in the same host were used in successive rounds of staining.

First, we began by optimization of avidin and biotin (A&B) blocking. The omission of the A&B blocking step resulted in extensive (~ 35%) cross-reactivity (Fig. 2A). By digital quantification, the area of staining that remained when A&B blocking was less than 1%. However, we found that the blocking was not uniform across the tissue. More cross-reactivity was seen in areas with very intense GFAP staining, which is problematic as reactive cells could be incorrectly phenotyped because of varied expression patterns of antibody staining. Therefore, we further optimized the A&B blocking step by varying the concentration of A&B in the blocking solution, increasing the volume of the blocking solution, and increasing the incubation times (Table 2). With the most rigorous A&B blocking method, we observed the greatest degree of blocking and minimal cross-reactivity. However, by the digital pathological quantification, all the conditions were equally effective (Fig. 2A, Table 2).

**Table 2:** Blocking step optimization

Trial	A&B (drops/ml)	Device	Time (min)	FAB (ug/ml)	Device	Time (hr)	Area fraction (%)	Qualitative evaluation
1	0	–	–	20	S	1	34.305	++++
2	4	S	15	20	S	1	0.066	+
3	6	L	15	20	S	1	0.009	+
4	6	S	60	20	S	1	0.001	–
5	6	L	15	20	S	1	0.005	+
6	6	L	60	20	S	1	0.003	–
7	10	S	15	20	S	1	0.245	+++
8	6	S	60	0	–	–	46.380	++++
9	6	S	60	20	S	1	0.004	+
10	6	S	60	40	S	1	0.007	+
11	6	S	60	40	S	18	0.003	–
12	6	S	60	40	L	1	0.006	+
13	6	S	60	40	L	18	0.009	–
14	6	S	60	60	S	1	0.098	+
15	6	S	60	100	S	1	0.133	++

During the incubations slides were in either Sequenza (S) or lockmailer (L) device. The HALO area fraction module was used to determine the % of stained tissue. An observe blind to the experimental conditions also rated the slides as robust residual staining (++++), some cells present throughout the tissue (+++), a few cells present in select regions (++), faint profiles for staining still observable (+), or no observable staining (–)

Next, we optimized the fragment antigen-binding region (FAB) blocking step. The omission of the FAB blocking step resulted in ~ 46% remaining cross-reactivity (Fig. 2B). As with A&B blocking, increasing concentrations of FAB increased blocking efficacy up to a concentration of 40 mg/ml (Table 2). When assessed with unbiased digital methods (HALO), there were few differences between blocking methods, regardless of incubation time or device used. However, visually lightly stained cells were present with shorter incubation and low reagent volume compared to the longer incubation times and higher volume reagents.

### Test of refined MICSSS protocol on antigenicity in human FFPE brain tissue

**Fig. 3:**
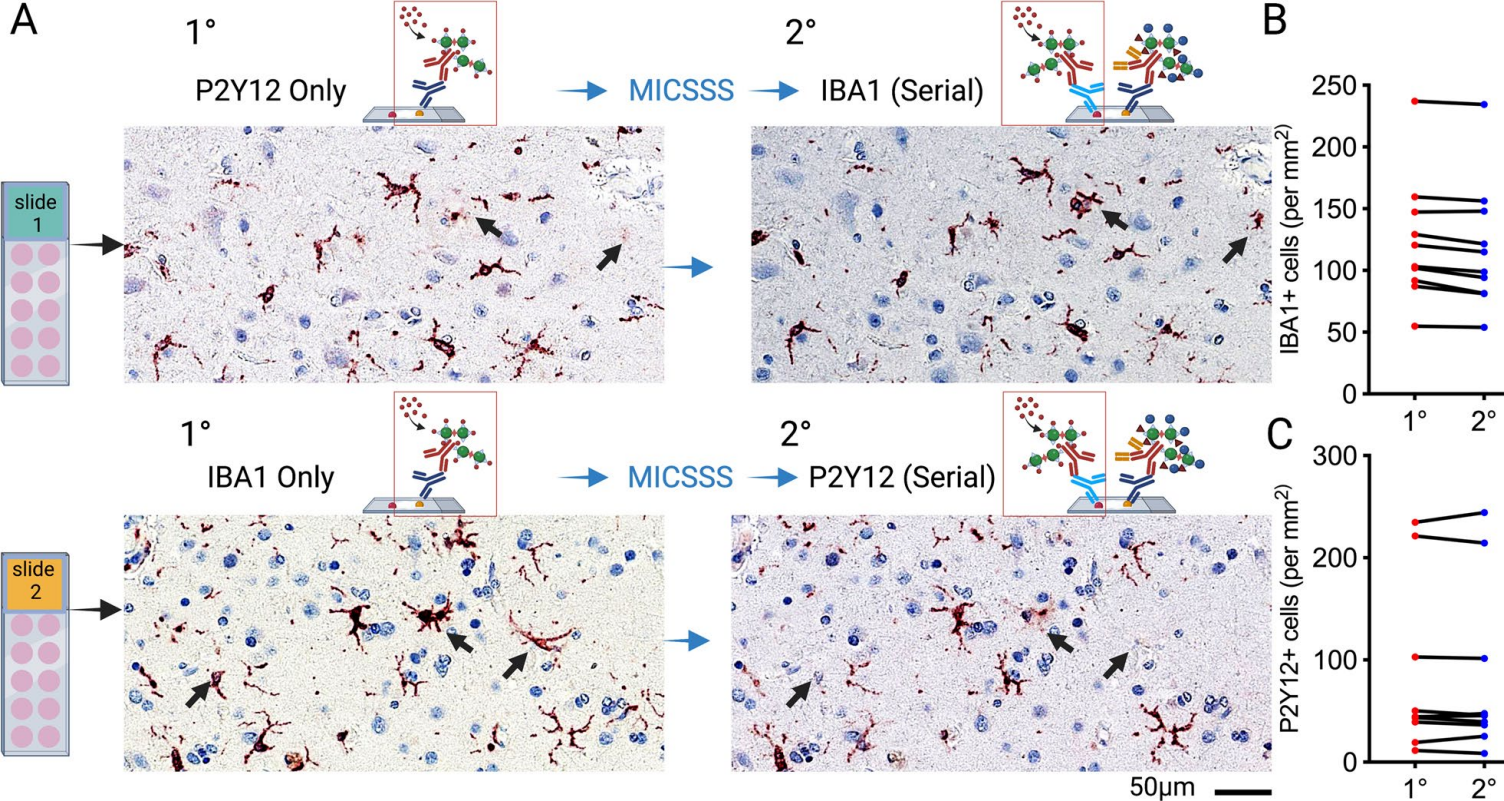
Effects of mIHC protocol on antigenicity following repeated rounds of staining. (**A**) Serial sections of a Tissue microarray containing human brain samples from individuals with ADRC-NC were stained with rabbit-anti-P2Y12 or guinea pig-anti-IBA1 for the first round (1°) of staining. Digitalizing the side was followed by the refined MICSSS protocol and a second (2°) round of staining. Arrows indicated IBA1^+^P2Y12^–^ cells. The number of cells in each case on the TMA was quantified using the object colocalization algorithm (HALO 3.4). By a paired t-test, no statistical difference was seen for between 1° or 2° rounds of staining the number of IBA1^+^ cells (**B**) or P2Y12^+^ cells

Remark et al. [9] previously showed, using serial sections of FFPE colorectal tumor tissue, that following even seven rounds of de-staining, there was no observable loss of antigenicity. Moreover, they found that they could stain for up to four macrophage markers with no observable steric hindrance. We tested if brain tissue and microglia markers would also be resistant to loss of antigenicity from the de-staining procedure or steric hindrance. Using serial sections from a tissue microarray (TMA) of human brain FFPE tissue, we stained the tissue with microglia markers IBA1 and P2Y12. While IBA1 is a pan-marker of microglia and macrophages, P2Y12 is a marker of homeostatic microglia. Therefore, we anticipated that most cells would be double positive for P2Y12 and IBA1. However, some fraction of the IBA1 positive cells should be P2Y12 negative. TMA slides were stained with IBA1 or P2Y12, followed by MICSSS, and then stained with the other antibody (Fig. 3A). As predicted, most of the stained cells were double positive for IBA1 and P2Y12. However, regardless if IBA1 was the first stain or second stain in the series, a fraction of the IBA1 positive cells were P2Y12 negative (Fig. 3A). Digital quantification of the number of cells using the object colocalization algorithm (HALO) showed variability in the number of IBA1 cells in the TMA brain sections from the ten different individuals, which would be expected as the cases had different degrees of pathology. However, for the same case, the number of IBA1 positive cells was steady between the first and second round of antibody staining (Fig. 3B). The P2Y12 antibody also stained a steady number of cells between rounds of staining (Fig. 3C), and a paired t-test showed no statical difference for IBA1 (Fig. 3B) or P2Y12 (Fig. 3C). These results provide additional evidence that the MICSSS protocol can work in brain FFPE tissue without loss of antigenicity.

### Spatial relationship of microglia to Aβ plaques and PHF-1^+^ tangles using multiplexed single-cell analysis

**Fig. 4:**
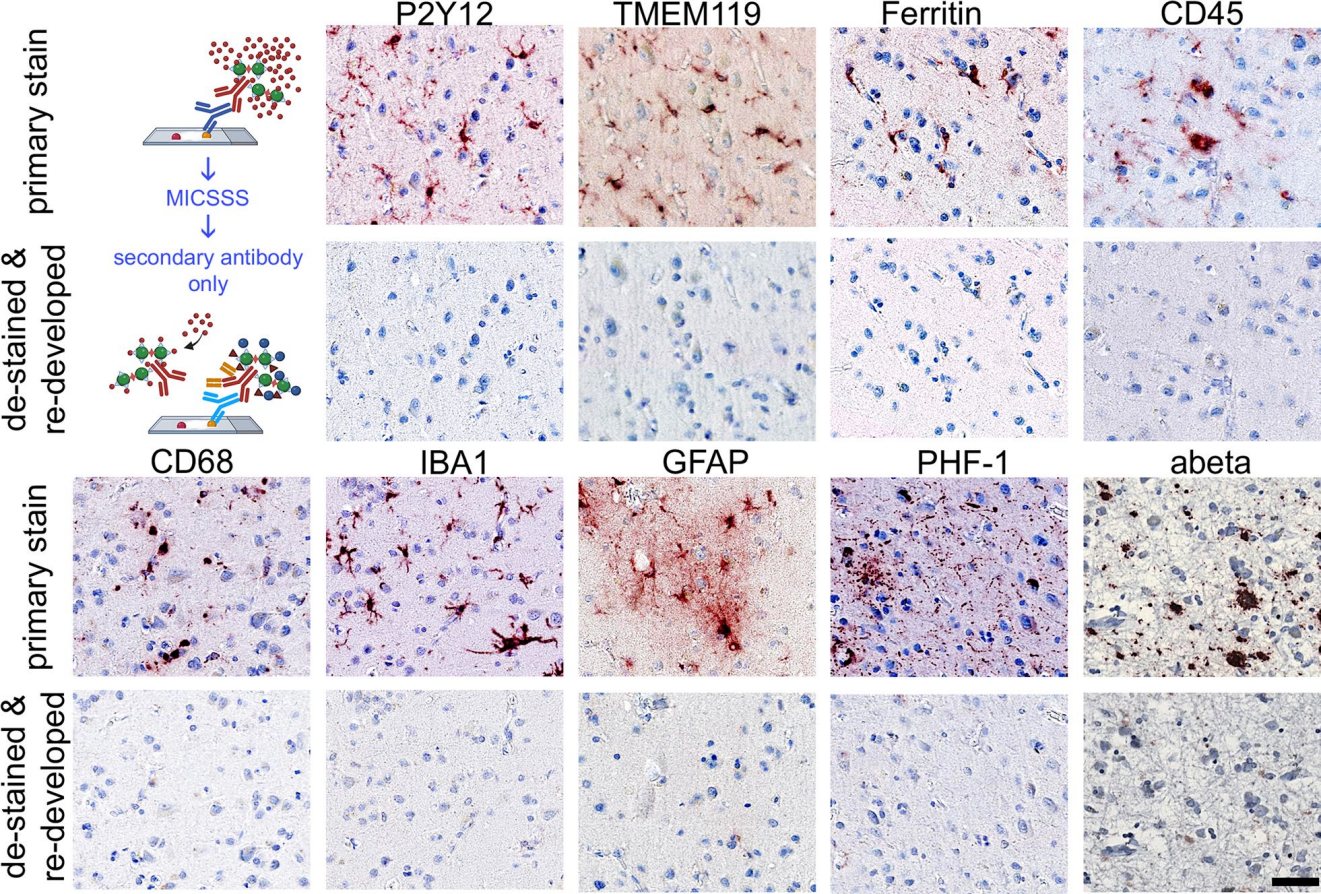
Panel of antibodies used from mIHC. FFPE human brain tissue was stained with antibodies used for the mIHC. The primary stain (1°) is shown, along with the image of the same section following the MICSSS de-staining, and then re-staining omitting the primary antibody. The order of antibody from left to right shows the order used on the mIHC panel. Using a HALO Area Quantification algorithm across the entire tissue section found, less than 0.03% of the primary stain, or other background noise, was detected in the de-stained and redeveloped tissue for all markers. Scale bar = 50 μm

**Fig. 5:**
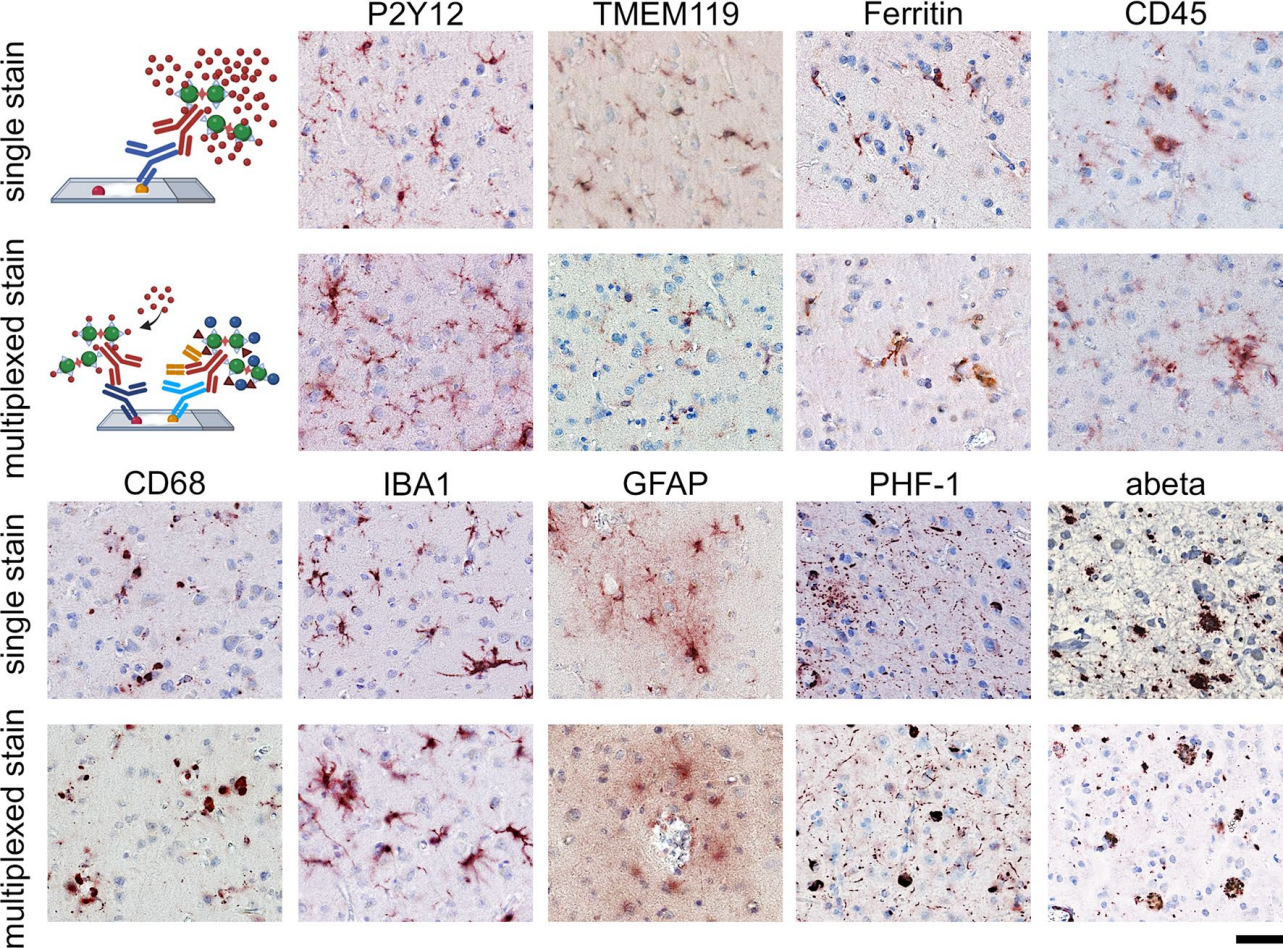
Comparison of single and sequential histological staining in glia-associated markers. Representative photomicrographs in similar regions from neighboring FFPE sections of the same sMTG tissue block, show comparative staining for the selected glial-associated stains. During each round of staining, tissue was stained alone as a positive control and sequentially using the multiplex staining method to show there is little to-no loss in antigenicity or stainability in subsequent rounds of staining. The difference in the area quantification of staining between the multiplex vs. single stain was 0.5% or less. The single stain and multiplex stain analysis was done on serial sections of the same sMTG tissue block; however, there may be a separation of up to 100 μm in the z dimension between sections. Photomicrographs were captured at × 10 magnification. Scalebar is 50 μm

AD lesions, including neurofibrillary tangles (NFT) comprised of abnormally phosphorylated tau protein and extracellular plaques containing amyloid-beta (Aβ) proteins, provide an excellent test to define the heterogeneity of microglia populations using histo-cytometry. We defined a panel of nine antibodies to test the spatial heterogeneity of microglia in relation to Aβ plaques and PHF-1-positive NFTs (Fig. 4). We showed that each antibody could each be de-stained and the cross-reactive was effectively blocked using the refined MICSSS protocol. Moreover, there was no apparent loss of antigenicity from the first to the ninth round of staining (Fig. 5).

**Fig. 6:**
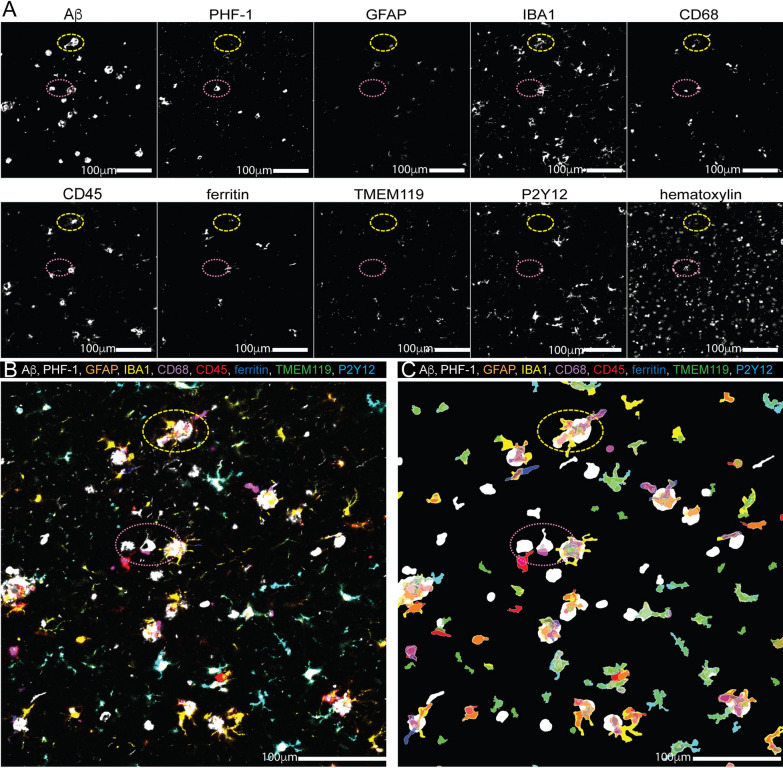
QUIVER image registry and cell identification. (**A**) Pseudocolored images were created from deconvolved single-channel IHC images. (**B**) The images were aligned using HALO software and generating a ten-channel image (nine antibodies and hematoxylin). (**C**) Cell/object count data, including marker co-expression, were generated using the object colocalization algorithm. Label colors coincide with representative color in markup image and merged image. Yellow dashed oval highlights a representative amyloid plaques and pink doted oval highlights a representative of tau tangles shown in all images

After validating that the antibodies in the panel could be effectively de-stained and antibody cross-reactivity could be blocked, we deconvolved the multiplex slide and registered the images using HALO software. Figure 5 shows the results of the spatially registered multiplex image. PHF-1 and Aβ were the last two stains completed in the multiplex protocol and clearly show microstructures with the expected morphological appearance of tangles and plaques, respectively (Fig. 6A−B).

The multiplex panel included six well-characterized antibodies representing different microglia/macrophage functional states. IBA1 (Fig. 6A) was included as a pan microglia/macrophage marker, although there are reports of IBA1-negative microglia [19, 20]. In this study, we used CD45 (Fig. 6A) to identify reactive microglia and macrophages. However, CD45 is a pan leukocyte marker, thus, is not specific to microglia/macrophages. P2Y12 (Fig. 6A) and TMEM119 (Fig. 6A) were included as markers for homeostatic microglia [21]. CD68 (Fig. 6A) and ferritin (Fig. 6A) were included as functional state markers associated with phagocytosis and iron storage, respectively [21]. Finally, GFAP (Fig. 6A) was included as a control marker to ensure that microglia/macrophage-marker-positive cells were not GFAP-positive. The merged imaged (Fig. 6B) illustrates the excellent registration of nine digital slides.

The next step in the spatial analysis workflow was to create histo-cytometric counts of the cells/objects in the mIHC image. To generate object counts, we used the Halo object colocalization algorithm to generate object counts (Fig. 6C). Size exclusions (40μm^2^) were included, so the algorithm only counted larger-size objects (i.e., cell bodies and plaques) to avoid counting PHF-1^+^ neurites and oblique cuts of the microglia process as a cell/object. Figure 6C shows the algorithm's results and highlights the heterogeneity in microglia in the human brain tissue.

**Fig. 7:**
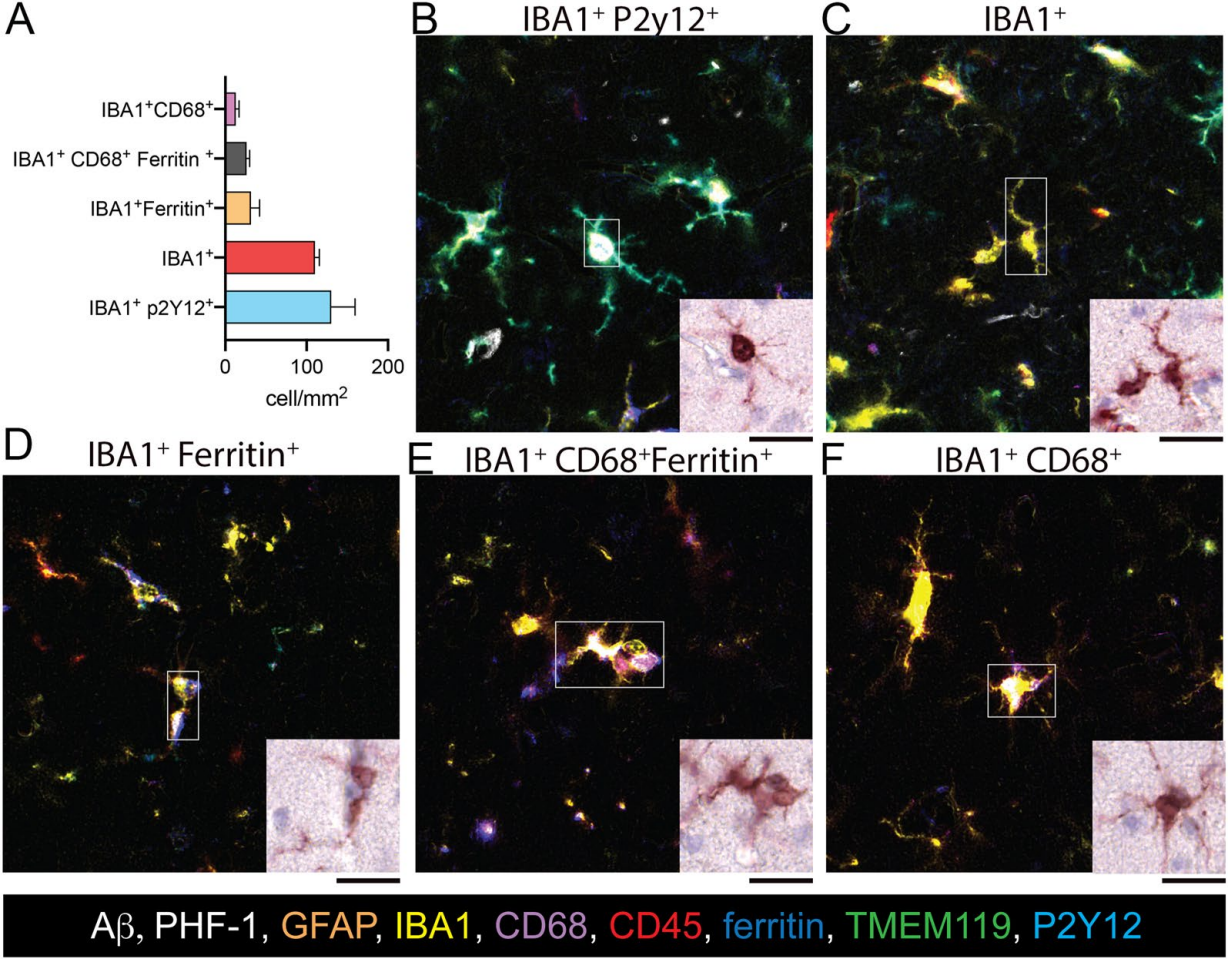
Identifying of microglia/macrophage phenotypes in human FFPE brain tissue using QUIVER. (**A**) Microglia/macrophages were grouped into one of five phenotypes based on unique marker expression. Human gray matter was analyzed to determine how many cells showed those phenotypes on average using the HALO object colocalization algorithm. (**B**–**F**) The pseudo-fluorescent images were created from deconvolved single-channel IHC images. IBA1 (yellow), P2Y12 (green), ferritin (blue), and CD68 (magenta) were included in the pseudo-fluorescent images. The white box indicates a cell that expressed the different marker classes, as determined by the HALO object colocalization algorithm. The other cells in the micrograph may not share the same cell phenotype. The original brightfield image of the IBA1 IHC is shown for the cell highlighted in the box to highlight differences and similarities in the IBA1^+^ cellular morphology among the molecular distinct microglia/macrophage phenotypes. Scale bar = 25 μm

Five unique IBA1^+^ microglia/macrophage phenotypes based on antibody marker expression were identified. The most prevalent cell type was the IBA1^+^ cells that express the homeostatic microglia marker P2Y12 (Fig. 7A, B). Somewhat unexpectedly, the second most abundant phenotype was cells expressing only IBA1 and none of the other microglia/macrophage markers (Fig. 7A, C). Finally, cells that expressed markers of macrophage/reactive microglia accounted for approximately a third of all IBA1^+^ cells. Interestingly, this third of the IBA1+ cells could be subdivided into the cells that expressed ferritin (Fig. 7A, D), ferritin and CD68 (Fig. 7A, E), and only CD68 (Fig. 7A, F).

TMEM119 and CD45 were found not useful markers for identifying molecularly unique microglia populations. Most TMEM119^+^ cells were also P2Y12^+^, but not all the P2Y12^+^ cells expressed TMEM119. In contrast, CD45 was present in most of the IBA1^+^ cells. However, the level of CD45 expression was low on P2Y12^+^ cells and high on amyloid-associated microglia. Including CD45 low vs. high did not help define a unique microglia population.

**Fig. 8:**
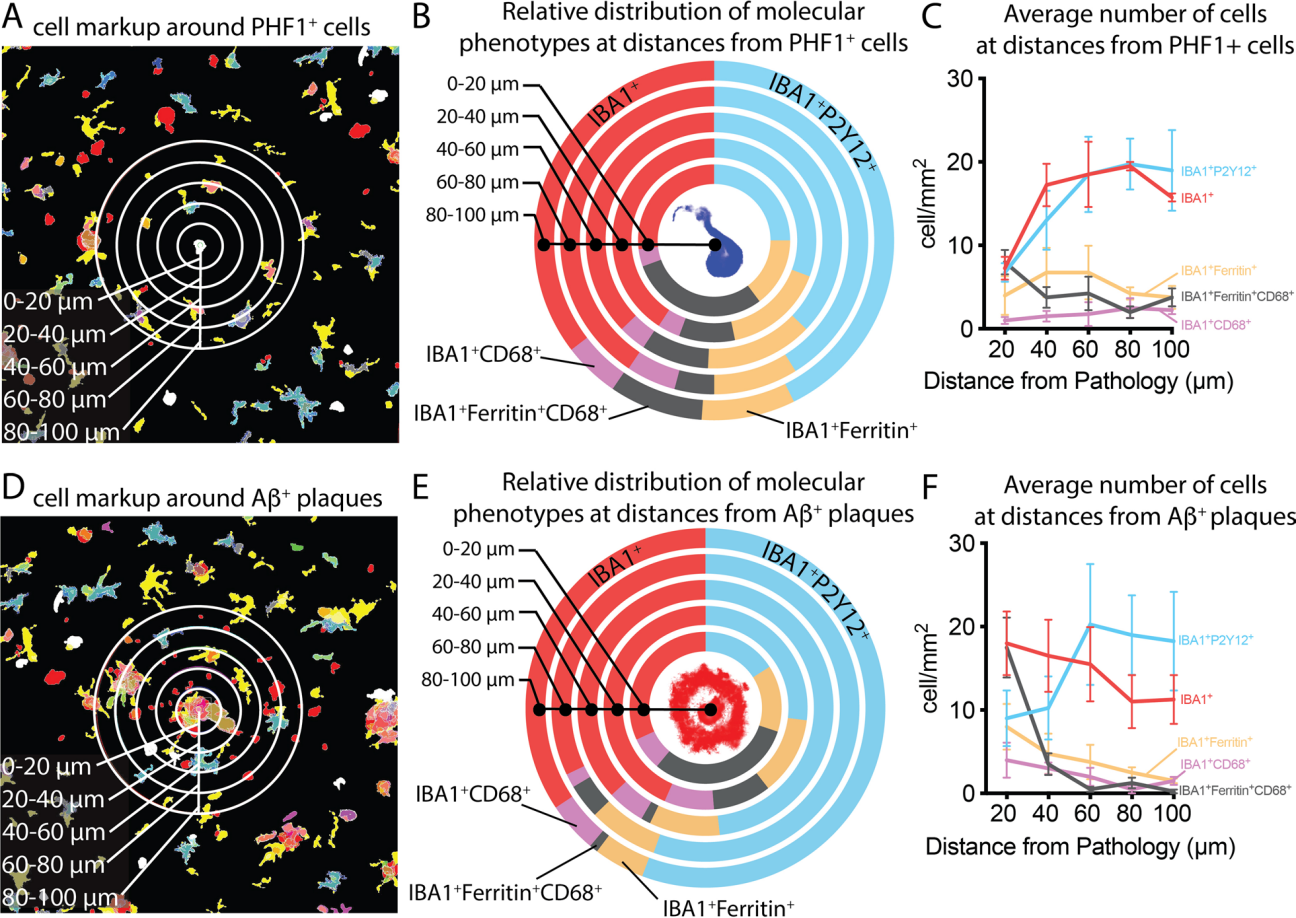
Digital proximity analysis of microglia/macrophage phenotypes to PHF-1^+^ tangles and Aβ^+^ plaques. (**A**) The Halo software generated markup shows the few cellular profiles within 60 μm of the PHF-1^+^ cell. (**B**) The Halo proximity analysis defined the relative percentage of the five microglia/macrophage phenotypes at each distance interval from the PHF-1^+^ cell. (**C**) The average number of cells at the distance intervals away from the PHF-1^+^ cell. (**D**) Near an Aβ^+^, plaque there is a high density of cells. (**E**) Nearest the plaque the IBA1 + Ferritin + CD68+ cell, and the IBA1+ phenotypes account for the majority of the plaque-associated cells. (**F**) The number of microglia/macrophage at the distance interval shows the polarization of marker expression that occurs around 50 μm from the plaque. The results are for 942 and 890 IBA1+ cells for figures A-C and D-F, respectively

We next asked if the spatial distribution of the five microglia/macrophage phenotypes was a function of the cell’s proximity to a PHF-1^+^ cell or an amyloid plaque. We used the HALO proximity analysis algorithm to determine the spatial distribution of the five microglia/macrophage phenotypes within a 100 μm radius from the PHF-1^+^ cell or Aβ^+^ plaque. As Aβ plaques and tangles are often in proximity to another plaque or tangle, the 100 μm radius was set to try to limit spatial overlap. The lowest density of microglia/macrophages occurred nearest to the PHF-1^+^ cell (Fig. 8A). Nearest to the PHF-1+ cell, few cells expressed homeostatic microglia markers (TMEM119 and P2Y12) (Fig. 8B). In contrast, most cells closest to the tangle expressed all the macrophage/reactive microglia markers (CD68 and ferritin) (Fig. 8B). Macrophage/microglia populations immunoreactive for IBA1 and Ferritin; IBA1, Ferritin, and CD68; IBA1 and Cd68 (further described as reactive macrophage/microglia phenotypes) showed little change in the number of cells at different distances from the PHF-1^+^ cells (Fig. 8C). In contrast, microglia not expressing CD68 and ferritin dramatically increased at a greater distance away from the PHF-1^+^ cells (Fig. 8C).

While cell density was lowest near the PHF-1^+^ cells, Microglia/macrophages were at the highest density around amyloid-plaques, and the density decreased nearly proportional to the distance from the plaque (Fig. 8D). Nearest the plaque, a high percentage of the cells expressed CD68 and ferritin (Fig. 8D). The increase in cells expressing the reactive markers, were proportional to the decline in homeostatic microglia (Fig. 8D). Interestingly, while few cells expressed only the homeostatic microglia markers within 40 μm of a plaque, this population rebounded beyond 60 μm from the plaque (Fig. 8F).

While the IBA1^+^Ferritin^+^CD68^+^ cells were most strongly associated with Aβ plaques, the IBA1^+^CD68^+^ cells did not have a clear spatial preference for plaque or tangle pathology. Therefore, we visually inspected each of the IBA1^+^CD68^+^ and IBA1^+^Ferritin^+^CD68^+^ using the mIHC image and the co-registered IBA1 brightfield IHC image (Fig. 8).

**Fig. 9:**
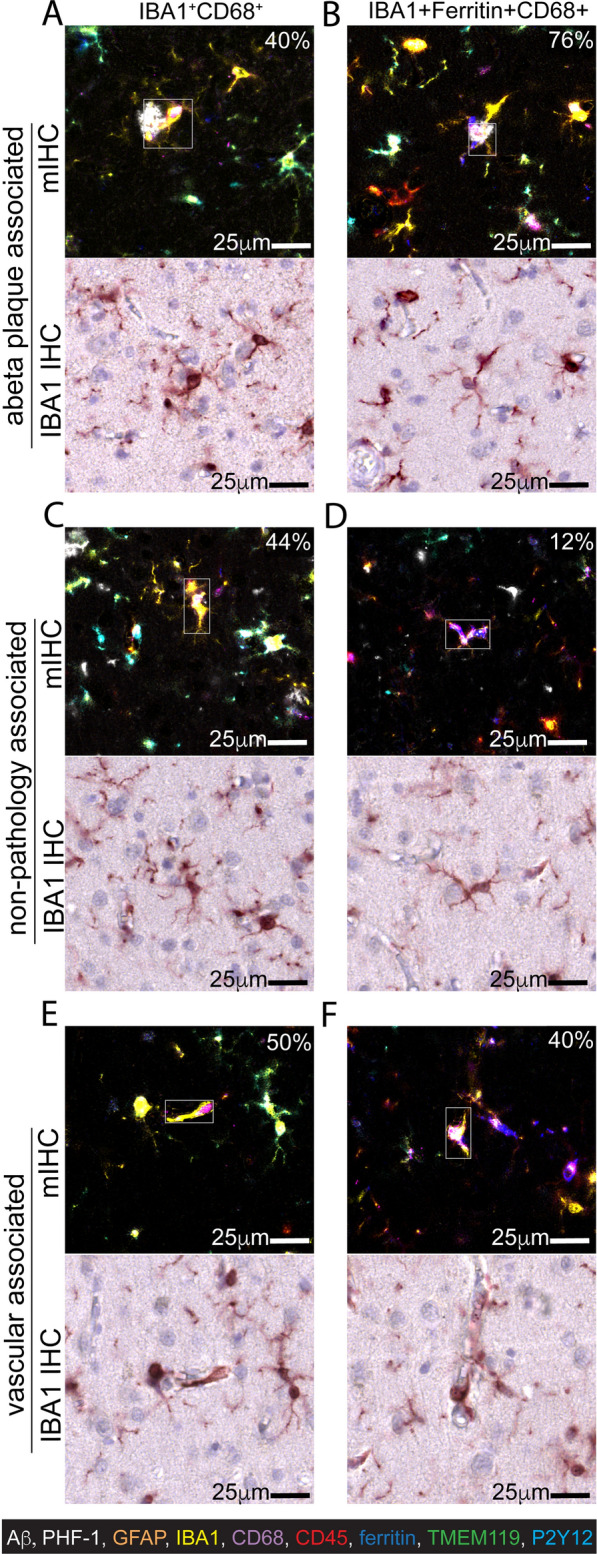
Spatial characterization of molecularly distinct microglia/macrophage phenotypes in relation to pathology and vascular profiles. A representative example of the nine-color multiplex IHC and single-color IBA1 IHC for IBA1^+^CD68^+^ (**A**, **C**, **E**) and IBA1^+^Ferritin^+^CD68^+^ (**B**, **D**, **F**) cells associated with Aβ plaques (**A**, **B**) non-plaque or PHF-1 associated (**C**, **D**), or vascular associated (**E**, **F**). The percent of the cells associated with pathology, non-pathology, or blood vessels is indicated on the image. A total of 1382 IBA1+ cells were included in the analysis

In agreement with the proximity analysis, IBA1^+^CD68^+^ cells (Fig. 9A) and the IBA1^+^Ferritin^+^CD68^+^ cells (Fig. 9B) were often associated with Aβ plaques. The IBA1^+^Ferritin^+^CD68^+^ cells were found touching an Aβ plaque 76% of the time, while 40% of the time, the IBA1^+^CD68^+^ cells were touching an Aβ plaque. IBA1 morphology was remarkably similar between these two cell types, showing both small cell bodies and thin branches (Fig. 9A, B).

A subset of the IBA1^+^CD68^+^ cells (Fig. 9C) and the IBA1^+^Ferritin^+^CD68^+^ cells (Fig. 9D) were not adjacent to Aβ plaques, or PHF-1 staining. Approximately half (44%) of the IBA1^+^CD68^+^ cells were not pathology associated. However, it was rare (12%) to find IBA1^+^Ferritin^+^CD68^+^ cells not near a plaque or tangle. Morphologically, there was no distinction between the two cell phenotypes, providing further evidence that morphology alone misses molecularly distinct microglia phenotypes.

We discovered that a significant proportion of plaque-associated and plaque-unassociated cells showed tight connections with vascular profiles during inspection. Therefore, we next counted the number of vascular-associated cells. 50% of IBA1^+^CD68^+^ cells (Fig. 9E) and 40% of IBA1^+^Ferritin^+^CD68^+^ cells (Fig. 9D) were border-associated macrophages.

## Discussion

Within, we report a new tool called QUIVER (Quantitative multiplex Immunohistochemistry with Visual colorimetric staining to Enhance Regional protein localization). With QUIVER, we sought to use conventional immunohistochemistry (IHC) techniques and digital pathological tools to expand the reach of spatial proteomics to more neuroscience researchers. We detail a multiplex IHC strategy using FFPE-preserved human brain tissue with neurodegenerative pathology. This technique successfully distinguishes between five subsets of microglia/macrophages by antibody marker expression and describes the relationship between these five phenotypes and amyloid plaques, NFT pathology and vasculature. We demonstrated a plausible transitional stage in IBA1^+^ cells, which lack homeostatic microglia but do not express the reactive markers CD68, and ferritin. A subset of microglia/macrophages expressing CD45, and ferritin were also identified, primarily associated with plaques. Finally, the work demonstrated that morphology did not strongly associate with molecular phenotypes, providing further evidence microglia morphology provides complementary information regarding cell stated that is distinct from molecular phenotype. These results demonstrate this technique’s power but are only a starting point. The multiplex IHC tool can move beyond defining well-known cell-type specific markers and evaluate pathways shared by multiple cells in the brain microenvironment. An example would be looking at the activation of signal transduction pathways in multiple kinds of cells. Although microglia were the primary focus of this investigation, the tools we report can be applied to any cell or protein target, including the interaction of multiple misfolded proteins within one cell.

Human brain tissues (particularly if extensive adjunct data are available) provides a unique resource that enables hypotheses to be tested that are directly relevant to clinical disease. Although FFPE-preserved tissue is the most widely available human biobanked tissue, it is not an unlimited resource. Therefore, identifying methods that can maximize the knowledge gained, while using the least possible amount of tissue, will have an outsized impact on expanding access to these valuable scientific specimens. Therefore, we set out to make a multiplexed chromogen-based IHC staining assay that can be easily used in most labs to meet the need for high-dimensional analysis of microglia in the context of neurodegenerative disease pathology. QUIVER uses digital pathology tools and does not require any extra equipment for staining or imaging. This makes it comparable in terms of cost and quality as standard IHC. These advancements will make it easier to categorize microglia into different phenotypes and providing additional levels to the microglia analysis in studies of neurodegenerative diseases and beyond.

One of the biggest challenges of any multiplex immunostaining of postmortem human FFPE brain tissue, particularly that from individuals that suffered an acute brain injury or from older individuals, is extensive autofluorescence. For example, lipofuscin, a normally occurring autofluorescent lipopigment, emission spectrum presents in a wide range of wavelengths, ranging from 400 to 700 nm thus interfering with the most commonly used fluorescent wavelengths [22–24]. While there are a number of reagents available (such as, sudan black or true black), that can quench the autofluorescence signal, the quenching is often incomplete. Lipofuscin autofluorescence is particularly problematic when the real staining is expected to be intracellular, as lipofuscin can erroneously be included as the antibody specific staining. For proteins of low abundance, it is often difficult to amplify the immunofluorescence signal over tissue background autofluorescence, even with the use of autofluorescence blocking reagents, and methods to amplify immunofluorescence staining, including the tyramide signal amplification system [25]. Finally, computational methods provide additional way to overcome autofluorescence signal [26]. Immunofluorescence staining is also not the standard method used in clinical pathology. QUIVER was developed as an approach that could use chromogen-based multiplexing to overcome many of these limitations.

A second major potential limitation of high-plex immunostaining methods is the limited number of donor host species for the primary antibody. Often, this means using suboptimal or less well-validated antibodies to avoid the same primary host antibody interaction. Alternatively, using primary antibodies directly conjugated to a reporter molecule is a common approach used in multiplex immune-staining methods to overcome the same primary antibody host limitation. Directly conjugated antibodies are widely available and used extensively (for example) in flow cytometry. It is, however, common for antibodies optimized for flow cytometry to be incompatible with FFPE tissue. Custom labeling antibodies is costly and requires a significant amount of starting material. Optimization would need to be completed on the custom antibody to determine the concentration needed for the staining. Alternatively, chemical antibody elution provides a method to remove the antibody, so an additional round of antibody staining can be applied. Our attempts at antibody stripping using the MILAN method were not successful. Some antibody elution is predicted by the HIER in the MICSSS method. However, we still found robust redevelopment if we did not include FAB or A&B blocking steps in the protocol, suggesting that the extent of antibody elution following HIER is minor.

Our research allowed using multiple same-species antibodies on tissue by blocking cross-reactivity with FAB and A&B blocking reagents at saturating concentrations. We also found that adjusting concentration, volume, and time of blocking reagents was critical to eliminate cross-reactivity. Also important is validating the lack of cross-reactivity in positive control tissue with robust / maximum expected expression of the antigen. After establishing our FAB and A&B blocking conditions on the anti-GFAP antibody, we found the same conditions were effective for all of our other stains. However, we also found for antibodies that did not stain as strongly as anti-GFAP, shorter blocking time and lower blocking reagent concentrations were also effective. Therefore, it is possible to save money and time by optimizing the blocking conditions of each antibody.

When designing the multiplex panel, it is important to consider the order that antibodies are applied. Steric hindrance and loss of antigenicity following multiple rounds of staining are possibilities. However, we and others, did not find loss of staining because of steric hindrance or loss of antigenicity. The most important consideration for the order of antibodies is any specialized antigen retrieval step. For instance, we completed the amyloid staining last, as we found the formic acid treatment used in the protocol destroyed the antigen for subsequent microglia membrane proteins. Therefore, confirming the compatibility of antigen retrieval step is critical for an effective multiplex IHC experiment.

Even though we specifically avoided immunofluorescence staining in the present set of experiments, others have described multiplex immunofluorescence methods using human FFPE tissue [27, 28]. Immunofluorescence staining is advantageous as it can drastically reduce the number of rounds of staining. The PICASSO method of ultra-multiplexed fluorescence imaging used spectral unmixing. In only three rounds of iterative staining, this approach achieved a remarkable 45-color image of the mouse brain [29]. However, the current version of the PICASSO method is computationally demanding, making it difficult to incorporate into most laboratories. In contrast, the iterative bleaching extends multiplexity (IBEX) method uses an approach of chemical bleaching of the fluorophore using LiBH4 [30, 31], similar to our methods making the transition between the mIHC and IBEX workflow seamless. A mixed approach of mIHC and IBEX- immunofluorescence could be particularly useful to increase throughput, where IBEX- immunofluorescence could be used for highly expressed antigens, while mIHC could be used for low expressed proteins that may masked by autofluorescence.

In addition to standard methods and tools that are widely available in many labs, this study used the HALO imaging suite as a unified tool for spatial analysis, visualization, and image registration. This proprietary software package's strength is its intuitive graphical user interface, which can be used by anyone, regardless of prior experience with programming. The HALO software environment is used currently in many basic sciences and clinical pathology labs. However, spatial proteomics is a rapidly developing field. Over twenty open-source imaging programs have been reported in the past five years. CellProfiler [32], histoCAT [33], CytoMAP [34], QuPath [35], and many others are examples of such programs. As a result, these rapidly developing open-source tools are driving innovations in spatial analysis. At the same time, proprietary software suites like HALO will likely need to continuously catch up in adopting the most recent advancements. Yet, while open-source software has driven innovation in genomics, it has created a bottleneck in data analysis.

In conclusion, we report a method for a refined multiplex IHC technique that can be used in biobanked human FFPE tissue. Using HALO digital pathological tools, we show the potential of spatial analysis to define unique subsets of microglia, which can be defined by proximity to pathology and marker expression, but not necessarily by the cellular morphology. The QUIVER will be a tool useful for better understanding the biologic implications of both the microglia transcriptomic data and the single-cell proteomic data. Through the use of conventional, low-cost reagents, this QUIVER may be broadly useful for the neuroscience community.

## Data Availability

The datasets used and/or analysed during the current study available from the corresponding author on reasonable request.

## Abbreviations

AD: Alzheimer’s disease
ADNC: AD neuropathological change
A&B: Avidin and biotin
FAB: Fragment antigen-binding region
FFPE: Formalin-fixed and paraffin embedded
GFAP: Glial fibrillary acidic protein
HEIR: Heat-induced antigen retrieval
IHC: Immunohistochemistry
IBA1: Ionized calcium binding adaptor molecule 1
mIHC: Multiplex IHC
MICSSS: Multiplexed immunohistochemical consecutive staining on a single slide
MILAN: Multiple iteractive labeling by antibody neodeposition
cytof: Single-cell mass cytometry
scRNA-seq: Single-cell RNA sequencing
TMA: Tissue microarray

## References

[1] Andreasson KI, Bachstetter AD, Colonna M, Ginhoux F, Holmes C, Lamb B, Landreth G, Lee DC, Low D, Lynch MA (2016). Targeting innate immunity for neurodegenerative disorders of the central nervous system. J Neurochem.

[2] Leng F, Edison P (2021). Neuroinflammation and microglial activation in Alzheimer disease: where do we go from here?. Nat Rev Neurol.

[3] Paolicelli RC, Sierra A, Stevens B, Tremblay ME, Aguzzi A, Ajami B, Amit I, Audinat E, Bechmann I, Bennett M (2022). Microglia states and nomenclature: a field at its crossroads. Neuron.

[4] Pimenova AA, Raj T, Goate AM (2018). Untangling genetic risk for Alzheimer's disease. Biol Psychiatry.

[5] De Smet F, Martinez AA, Bosisio FM (2021). Next-generation pathology by multiplexed immunohistochemistry. Trends Biochem Sci.

[6] Lewis SM, Asselin-Labat ML, Nguyen Q, Berthelet J, Tan X, Wimmer VC, Merino D, Rogers KL, Naik SH (2021). Spatial omics and multiplexed imaging to explore cancer biology. Nat Methods.

[7] Berry S, Giraldo NA, Green BF, Cottrell TR, Stein JE, Engle EL, Xu HY, Ogurtsova A, Roberts C, Wang D (2021). Analysis of multispectral imaging with the AstroPath platform informs efficacy of PD-1 blockade. Science.

[8] Boisson A, Noel G, Saiselet M, Rodrigues-Vitoria J, Thomas N, Fontsa ML, Sofronii D, Naveaux C, Duvillier H, Craciun L (2021). Fluorescent multiplex immunohistochemistry coupled with other state-of-the-art techniques to systematically characterize the tumor immune microenvironment. Front Mol Biosci.

[9] Remark R, Merghoub T, Grabe N, Litjens G, Damotte D, Wolchok JD, Merad M, Gnjatic S (2016). In-depth tissue profiling using multiplexed immunohistochemical consecutive staining on single slide. Sci Immunol.

[10] Wharton KA, Wood D, Manesse M, Maclean KH, Leiss F, Zuraw A (2021). Tissue multiplex analyte detection in anatomic pathology—pathways to clinical implementation. Front Mol Biosci.

[11] Keren-Shaul H, Spinrad A, Weiner A, Matcovitch-Natan O, Dvir-Szternfeld R, Ulland TK, David E, Baruch K, Lara-Astaiso D, Toth B (2017). A unique microglia type associated with restricting development of Alzheimer's disease. Cell.

[12] Schmitt FA, Nelson PT, Abner E, Scheff S, Jicha GA, Smith C, Cooper G, Mendiondo M, Danner DD, Van Eldik LJ (2012). University of Kentucky Sanders-Brown healthy brain aging volunteers: donor characteristics, procedures and neuropathology. Curr Alzheimer Res.

[13] Goltsev Y, Samusik N, Kennedy-Darling J, Bhate S, Hale M, Vazquez G, Black S, Nolan GP (2018). Deep profiling of mouse splenic architecture with CODEX multiplexed imaging. Cell.

[14] Coons AH, Creech HJ, Jones RN (1941). Immunological properties of an antibody containing a fluorescent group. Proc Soc Exp Biol Med.

[15] Bolognesi MM, Manzoni M, Scalia CR, Zannella S, Bosisio FM, Faretta M, Cattoretti G (2017). Multiplex staining by sequential immunostaining and antibody removal on routine tissue sections. J Histochem Cytochem.

[16] Gendusa R, Scalia CR, Buscone S, Cattoretti G (2014). Elution of high-affinity (>10-9 KD) antibodies from tissue sections: clues to the molecular mechanism and use in sequential immunostaining. J Histochem Cytochem.

[17] Middeldorp J, Hol EM (2011). GFAP in health and disease. Prog Neurobiol.

[18] Kenkhuis B, Somarakis A, Kleindouwel LRT, van Roon-Mom WMC, Hollt T, van der Weerd L (2022). Co-expression patterns of microglia markers Iba1, TMEM119 and P2RY12 in Alzheimer's disease. Neurobiol Dis.

[19] Lier J, Streit WJ, Bechmann I (2021). Beyond activation: characterizing microglial functional phenotypes. Cells.

[20] Lier J, Winter K, Bleher J, Grammig J, Mueller WC, Streit W, Bechmann I (2019). Loss of IBA1-Expression in brains from individuals with obesity and hepatic dysfunction. Brain Res.

[21] Hopperton KE, Mohammad D, Trepanier MO, Giuliano V, Bazinet RP (2018). Markers of microglia in post-mortem brain samples from patients with Alzheimer's disease: a systematic review. Mol Psychiatry.

[22] Di Guardo G (2015). Lipofuscin, lipofuscin-like pigments and autofluorescence. Eur J Histochem.

[23] Moreno-Garcia A, Kun A, Calero O, Medina M, Calero M (2018). An overview of the role of lipofuscin in age-related neurodegeneration. Front Neurosci.

[24] Schnell SA, Staines WA, Wessendorf MW (1999). Reduction of lipofuscin-like autofluorescence in fluorescently labeled tissue. J Histochem Cytochem.

[25] Stack EC, Wang C, Roman KA, Hoyt CC (2014). Multiplexed immunohistochemistry, imaging, and quantitation: a review, with an assessment of Tyramide signal amplification, multispectral imaging and multiplex analysis. Methods.

[26] Baharlou H, Canete NP, Bertram KM, Sandgren KJ, Cunningham AL, Harman AN, Patrick E (2021). AFid: a tool for automated identification and exclusion of autofluorescent objects from microscopy images. Bioinformatics.

[27] Ehrenberg AJ, Morales DO, Piergies AMH, Li SH, Tejedor JS, Mladinov M, Mulder J, Grinberg LT (2020). A manual multiplex immunofluorescence method for investigating neurodegenerative diseases. J Neurosci Methods.

[28] Liao RJ, Mondal M, Nazaroff CD, Mastroeni D, Coleman PD, Labaer J, Guo J (2021). Highly sensitive and multiplexed protein imaging with cleavable fluorescent tyramide reveals human neuronal heterogeneity. Front Cell Dev Biol.

[29] Seo J, Sim Y, Kim J, Kim H, Cho I, Nam H, Yoon YG, Chang JB (2022). PICASSO allows ultra-multiplexed fluorescence imaging of spatially overlapping proteins without reference spectra measurements. Nat Commun.

[30] Radtke AJ, Chu CJ, Yaniv Z, Yao L, Marr J, Beuschel RT, Ichise H, Gola A, Kabat J, Lowekamp B (2022). IBEX: an iterative immunolabeling and chemical bleaching method for high-content imaging of diverse tissues. Nat Protoc.

[31] Radtke AJ, Kandov E, Lowekamp B, Speranza E, Chu CJ, Gola A, Thakur N, Shih R, Yao L, Yaniv ZR (2020). IBEX: a versatile multiplex optical imaging approach for deep phenotyping and spatial analysis of cells in complex tissues. Proc Natl Acad Sci USA.

[32] Carpenter AE, Jones TR, Lamprecht MR, Clarke C, Kang IH, Friman O, Guertin DA, Chang JH, Lindquist RA, Moffat J (2006). Cell Profiler: image analysis software for identifying and quantifying cell phenotypes. Genome Biol.

[33] Schapiro D, Jackson HW, Raghuraman S, Fischer JR, Zanotelli VRT, Schulz D, Giesen C, Catena R, Varga Z, Bodenmiller B (2017). histoCAT: analysis of cell phenotypes and interactions in multiplex image cytometry data. Nat Methods.

[34] Stoltzfus CR, Filipek J, Gern BH, Olin BE, Leal JM, Wu YJ, Lyons-Cohen MR, Huang JY, Paz-Stoltzfus CL, Plumlee CR (2020). CytoMAP: a spatial analysis toolbox reveals features of myeloid cell organization in lymphoid tissues. Cell Rep.

[35] Bankhead P, Loughrey MB, Fernandez JA, Dombrowski Y, McArt DG, Dunne PD, McQuaid S, Gray RT, Murray LJ, Coleman HG (2017). QuPath: open source software for digital pathology image analysis. Sci Rep.

